# COVID-19 Infection in a Patient With Fragile-X Syndrome

**DOI:** 10.7759/cureus.11266

**Published:** 2020-10-30

**Authors:** Jeremy D Kleiman, Karthik Veerapaneni, Javier Escovar, Jose Orsini

**Affiliations:** 1 Department of Medicine, St. George's University School of Medicine, Great River, USA; 2 Department of Medicine, The Brooklyn Hospital Center, Brooklyn, USA

**Keywords:** covid-19, fragile x syndrome, pulmonary critical care, cytokine storm syndrome, cytokine release syndrome (crs), sars-cov-2

## Abstract

Over the past several months we have learned about how pre-existent comorbidities may influence the outcome of patients infected with the coronavirus disease 2019 (COVID-19), with diabetes mellitus and obesity being commonly reported in association with increased mortality among these patients. In this report, the authors explore the case of a patient with Fragile-X Syndrome (FXS) who developed pneumonia due to COVID-19. FXS is an inherited cause of intellectual disability with potential neurologic and physiologic sequelae. In this narrative, we aim to describe how FXS may potentially play a role in the clinical course of patients with COVID-19.

## Introduction

Because of the novelty of severe acute respiratory syndrome coronavirus 2 (SARS-CoV-2), there has been insufficient literature providing evidence-based medicine guidelines for the management of patients infected with the virus. When the coronavirus disease 2019 (COVID-19) pandemic hit in late 2019 and early 2020, The Society of Critical Care Medicine (SCCM) and The Infectious Diseases Society of America (IDSA) endorsed provisionary guidelines in order to provide clinical guidance during the pandemic. The next several months witnessed the documentation of many cases, and the publication of innumerable studies providing important data on the disease and its management. There have been large, composite studies out of both China and the United States on the association between several comorbidities and the clinical course and outcomes of COVID-19 patients [1,2]. One comorbidity, of which there are no reports documented or analyzed, is that of COVID-19 patients with Fragile-X Syndrome (FXS).

FXS is a trinucleotide repeat disorder, and the most common inherited form of intellectual disability with a prevalence estimated at 1/4,000 in males and 1/5,000 - 8,000 in females worldwide [3,4]. This is a gender-linked disorder, which may present with behavioral features and poor language development similar to that seen in autism spectrum disorder [3]. It is not commonly seen as an immune disorder, but there is literature that supports immune dysregulation and decreased cytokine responses in patients with increased trinucleotide repeats such as those found in FXS [5,6]. This report aims to document a case of COVID-19 in a female patient with FXS, and examine any role this genetic disorder may have had in her clinical course and outcome.

## Case presentation

A 46-year-old female presented to the emergency department with a one-week history of worsening dyspnea on exertion, with chest tightness and fatigue. She denied any chest pain, fever, chills, nausea, vomiting, or diarrhea. She had multiple comorbidities including hypertension, morbid obesity, type II diabetes mellitus, and asthma, and had a past medical history of a deep venous thrombosis in her left lower extremity. Additionally, she was born with FXS. She was afebrile, tachycardic with a heart rate of 112/min, hypotensive with a blood pressure of 90/56, mildly tachypneic with a respiratory rate of 22/min, and with an oxygen saturation by pulse oximetry of 93% while breathing ambient air. On physical examination she was alert and oriented x 3, speaking in full sentences, not using accessory muscles of respiration, and there were no rhonchi/rales heard on bilateral lung auscultation. She was admitted to the General Medicine ward with the presumptive diagnosis of community-acquired pneumonia and possible pulmonary embolism. Laboratory values included a ferritin level of 272 ng/mL (10-204), a white blood cell (WBC) count of 11,300 cells/mm^3^ (4.8-10.8), a lactate dehydrogenase level of 333 U/L (125-220), a C-reactive protein of 94.84 mg/L (<5.00), and a creatinine level of 1.7 mg/dL (0.6-1.1). Arterial blood gas (ABG) showed a pH of 7.41 (7.35-7.45), a PaCO_2_ of 29 mmHg (35-48), and a PaO_2_ of 105 mmHg (83-108), while the patient was receiving oxygen therapy at 2 liters by nasal cannula.

**Figure 1:**
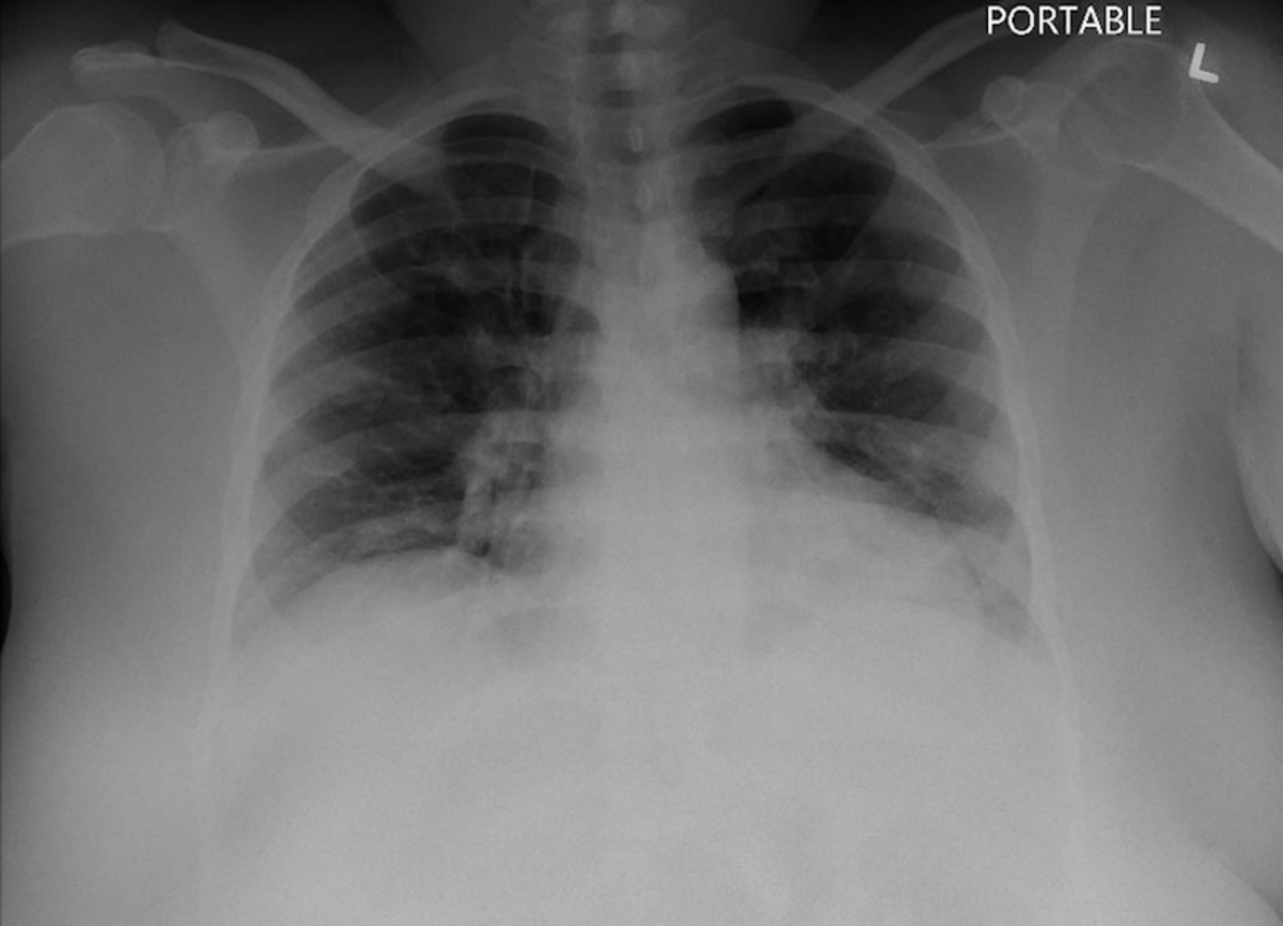
Chest radiography (CXR) from admission showing mild bilateral increased interstitial markings with no opacities or consolidations.

**Figure 2:**
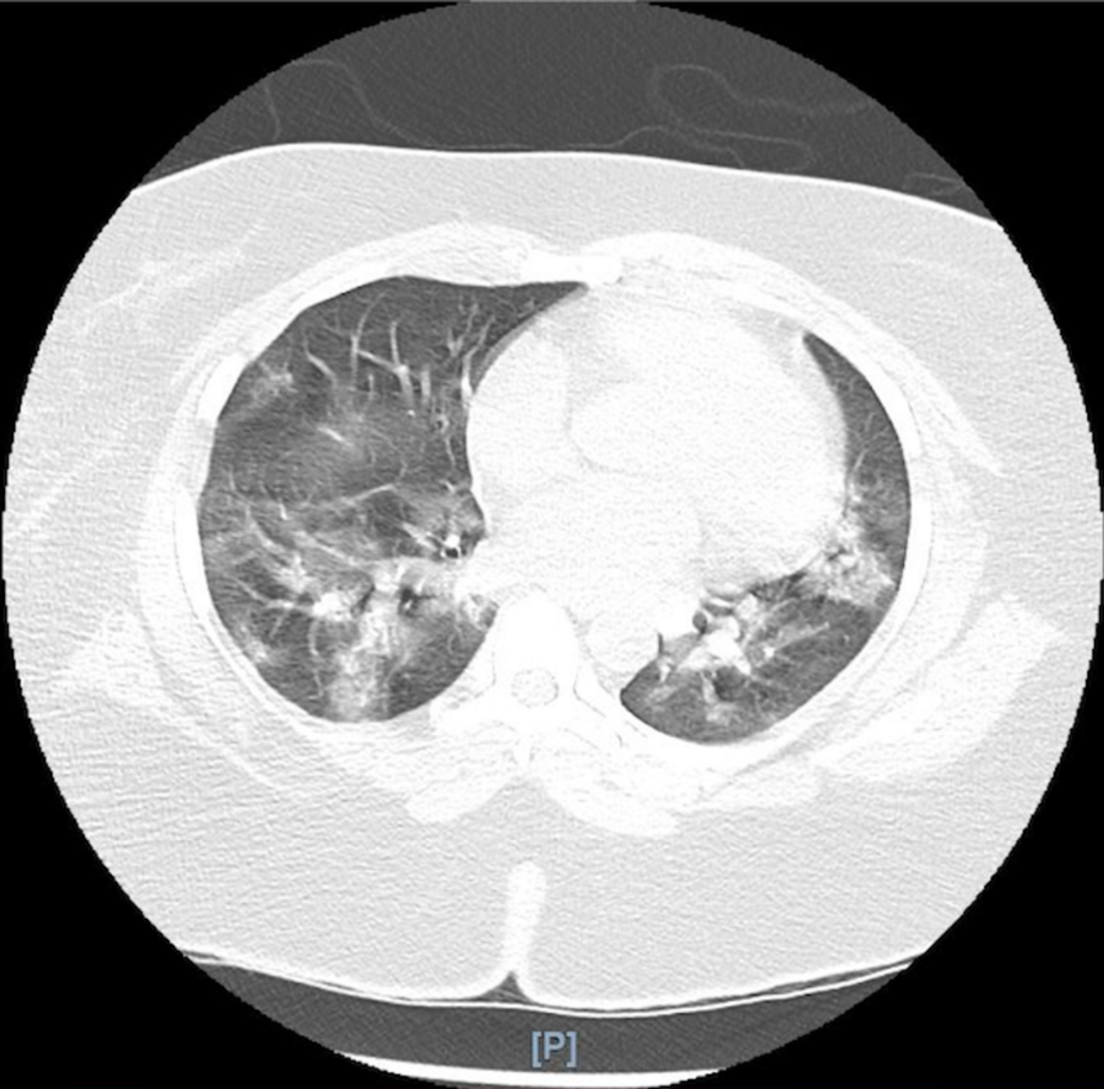
Chest CT without contrast demonstrating patchy, bilateral ground-glass opacifications.

Blood and respiratory cultures drawn on admission were negative. Serology and nasopharyngeal swabs for *Influenzae *A and B were negative. A polymerase chain reaction (PCR) of a nasopharyngeal swab for SARS-CoV-2 was positive. Initial chest radiography (CXR) showed no opacities or consolidations (Figure 1). A chest computed tomography (CT) without contrast showed patchy bilateral ground-glass opacities (Figure 2). Additionally, a doppler ultrasound of lower extremities demonstrated an acute deep venous thrombosis of the left lower extremity. She was treated empirically with ceftriaxone 1 g intravenously daily and doxycycline 100 mg intravenously every 12 hours, in addition to hydroxychloroquine 400 mg orally daily and enoxaparin 50 mg subcutaneously every 12 hours.

**Figure 3:**
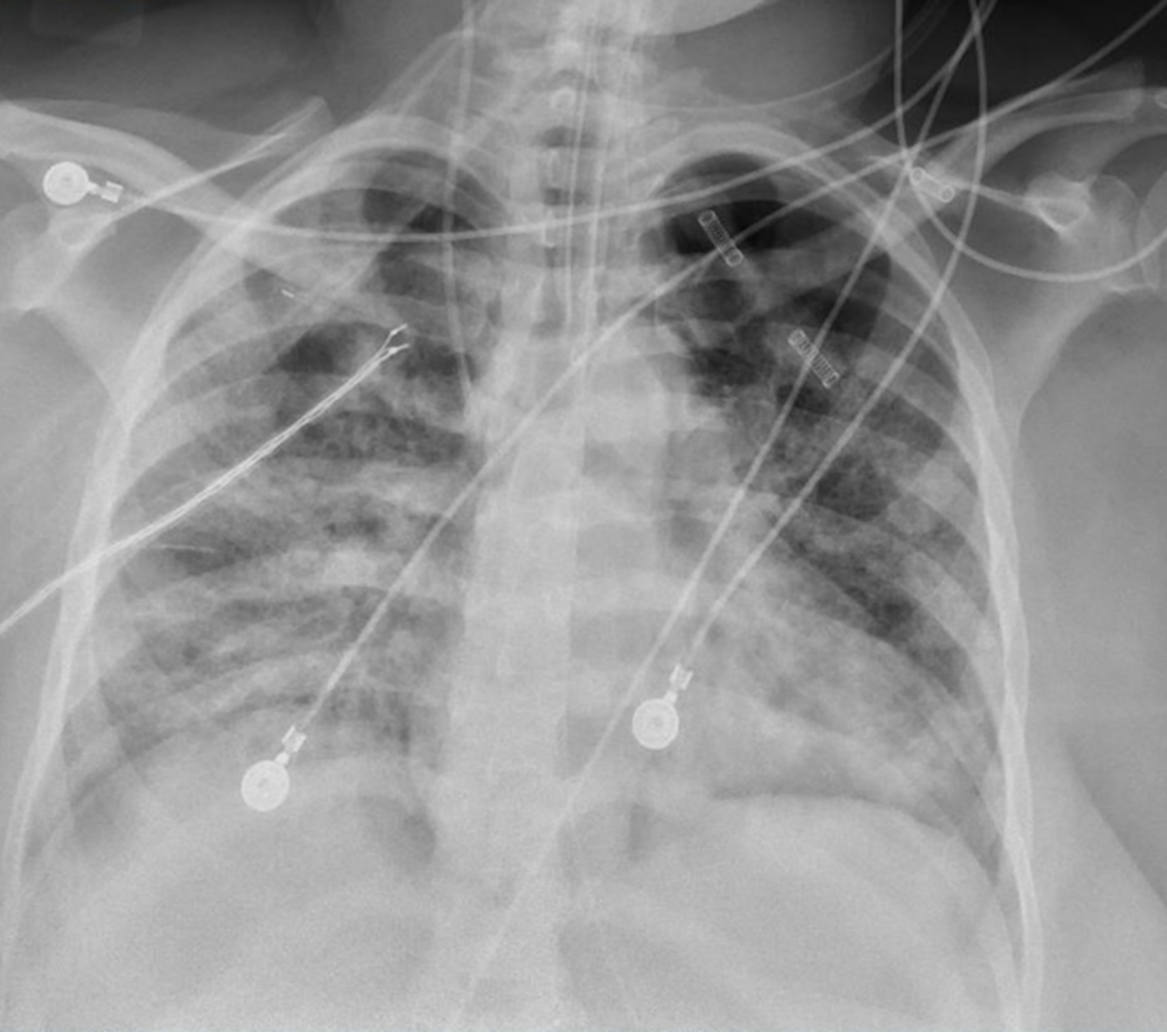
Chest radiography (CXR) on hospital day five showed diffuse heterogeneous airspace opacifications.

Because of worsening tachypnea and increasing oxygen requirements she was upgraded to the medical intensive care unit (MICU) on hospital day five, where she required intubation and mechanical ventilation. A repeat CXR showed patchy ground-glass opacities throughout both lung fields (Figure 3). Given the patient’s persistently elevated oxygen requirements as well as worsening CXR, intravenous tocilizumab, steroids (methylprednisolone 40 mg intravenously every 12 hours), and convalescent plasma infusion were administered as a salvage therapy for *Coronavirus*-associated pneumonia. Her prolonged ICU stay was complicated by shock requiring vasoactive therapy, ventilator-associated pneumonia with multi-drug resistant *Pseudomonas aeruginosa*, bacteremia with extended-spectrum β-lactamase (ESBL) producing *Klebsiella pneumoniae*, fungemia with *Candida auris*, acute kidney injury, and decreased hemoglobin levels as a consequence of a gluteal hematoma resulting from continuous heparin infusion. She failed multiple spontaneous breathing trials because of decreased mentation and a new-onset critical illness polyneuropathy and myopathy, and a tracheostomy was performed on ICU day 17. She was subsequently transferred to a step-down unit before being discharged to a long-term acute care facility.

## Discussion

To the best of our knowledge, this is the first reported case of COVID-19 in a patient with FXS. A recent study showed that FXS does not only affect the central nervous system but additionally causes other physiologic dysfunctions including changes in immune-related biomarkers. The study showed that patients with FXS have reduced serum levels of several chemokines including chemokine (c-x-c motif) ligand 10 (CXCL-10), which is a pro-inflammatory cytokine [7]. Therefore, a person with FXS may have impaired immunity and may be more susceptible to infectious diseases. CXCL-10 has also been implicated in the cytokine storm linked to a more severe disease among patients with COVID-19 [6,8,9]. Consequently, one hypothesis could be that patients with FXS infected with COVID-19 should display a lesser degree of inflammation and milder disease.

Cytokine Storm Syndrome or Cytokine Release Syndrome is a clinical entity characterized by a massive release of pro- and anti-inflammatory mediators by lymphocytes and macrophages that leads to uncontrolled local and systemic inflammation [9,10]. In patients infected with SARS-CoV-2, current literature suggests that release of pro-inflammatory cytokines by macrophages and monocytes leads to activation of T-lymphocytes and, ultimately, to a cascade of massive cytokine and chemokine production [9-11]^. ^This inflammatory cascade leads to a leaky vasculature and migration of white blood cells (WBCs) into tissues throughout the body, particularly the lungs resulting in acute lung injury[10,12,13]. Besides CXCL-10, some other cytokines determined to be of importance in the pathogenesis of COVID-19 are interleukin (IL)-1, IL-6, IL-10, tumor necrosis factor-alpha (TNF-a), interferon gamma (IFN-g), chemokine (c-c motif) ligand 2 (CCL-2), CXCL-9, and IL-8 [6,9,14-16].

Therefore, we hypothesized that the lowered CXCL-10 profile in patients with FXS would suggest a protective effect against cytokine release and Cytokine Storm Syndrome in patients with COVID-19. In this case, it is difficult to validate this hypothesis based on this patient’s tumultuous ICU course.

In addition, the patient described in this report had many comorbidities, including type II diabetes mellitus and morbid obesity, which are associated with a more severe clinical course and unfavorable outcome in patients with COVID-19 [1,2,17]. Furthermore, the patient discussed in this manuscript encountered many complications, including deep venous thrombosis and superimposed bacterial infections with multidrug-resistant organisms.

One of the limitations of this report is that we did not obtain serum levels of CXCL-10 or any other cytokines or chemokines. Because of the limited data on FXS patients with COVID-19, and still relatively limited knowledge on the cytokines involved in the pathophysiology of SARS-CoV-2 infection, it is difficult to draw any conclusions on the relationship between FXS and its protective effect against severe clinical courses in patients with COVID-19.

## Conclusions

Reduction in serum cytokine levels in patients with FXS may have a protective effect against the extensive inflammation involved in severe cases of COVID-19. As more literature becomes available, and the extent of cytokine involvement in the pathophysiology of COVID-19 is better understood, we will develop a better comprehension of the role of FXS in the clinical course and outcome of patients with COVID-19.
